# 

**Published:** 1883-05

**Authors:** 


CONTENTS.

| | Page. |
|---|---|
| Methods of Building up Health | 105 |
| Pure Air and Pure Blood | 107 |
| Piles | 115 |
| The Family Table | 116 |
| The Scarlet Fever | 117 |
| How to be Young at Eighty | 118 |
| Cheerful People | 118 |
| The Efficacy of Vaccination | 119 |
| Food Adulteration | 122 |
| Sleep | 123 |
| Inheritance | 124 |

